# Biochemical and Radiological Factors for Prognostication of Traumatic Brain Injury: An Institutional Experience

**DOI:** 10.7759/cureus.40999

**Published:** 2023-06-26

**Authors:** Abinav Sivashankar S, Sai Sriram Swamiyappan, Vivek Visweswaran, Rav Tej Bathala, Visvanathan Krishnaswamy, Venkata Shashank Davuluri, Ashwin Sridhar, Ganesh K

**Affiliations:** 1 Neurological Surgery, Sri Ramachandra Institute of Higher Education and Research, Chennai, IND; 2 Neurosurgery, Sri Ramachandra Institute of Higher Education and Research, Chennai, IND

**Keywords:** gcs (glasgow coma score ), brain ct scan, lactate dehydrogenase (ldh), serum cortisol, traumatic brain injury

## Abstract

Introduction

Traumatic brain injury (TBI) necessitates identifying patients at risk of fatal outcomes. Classic biomarkers used clinically today in other organ systems are quantitative in nature. This aspect largely restricts the prognostic ability of a theoretical quantitative brain biomarker. This study aimed to explore biochemical markers and imaging findings reflecting the severity of cerebral damage to predict outcomes.

Methodology

In this study, 61 TBI cases with moderate to severe brain injury were prospectively observed, and various indices including random blood sugar (RBS), hemoglobin, international normalized ratio (INR), lactate dehydrogenase (LDH), cortisol, and CT findings were assessed. Glasgow Outcome Scores (GOS) determined the outcomes. Statistical analysis was carried out to assess correlations.

Results

The mean RBS level of those who did not survive was 259.58 mg/dL, whereas in those who survived the value was 158.48 mg/dL. Analysis indicated that patients with high RBS value on admission had a higher risk of mortality (p=0.000). We noted that the mean serum cortisol levesl on both Days 1 and 5 were higher in patients who died and were able to establish a statistically significant correlation between both the values and outcome. A statistically significant negative correlation between Day 1 and Day 5 serum LDH levels and outcomes was evident from our study (p=0.000 for both). Among the components of the Rotterdam score, the presence of intraventricular hemorrhage (IVH) in the CT scan had a significant association with unfavorable outcomes (p=0.01) while midline shift was significantly associated with a low GCS (p=0.04).

Conclusion

Biochemical markers such as INR, RBS, serum cortisol, and LDH at admission can serve as valuable indicators of prognosis in TBI patients. Furthermore, a persistent increase in LDH and cortisol levels between Days 1 and 5, along with the Glasgow Coma Scale and Rotterdam Scoring system, are good predictors of mortality.

## Introduction

Trauma is the leading cause of death in people younger than 45 years of age. Traumatic brain injury (TBI) is the main cause of morbidity, disability, and mortality in this age group [1] and is referred to as a silent epidemic [2]. Severe TBI is associated with a 30-70% mortality rate with survival marked by severe neurological sequelae and impaired quality of life [3,4]. Glasgow Coma Scale (GCS) helps to assess the severity of the injury [4], while computed tomography (CT) scan remains the mainstay of imaging, with several scoring systems available to prognosticate outcomes [5].

TBI is associated with various metabolic and systemic changes, complicating the identification of patients with a risk of fatal outcomes [6]. The blood-brain barrier is a selective barrier that hinders most molecules from passing over its tightly maintained integrity. Assessing issues with the brain is more of a qualitative process than a quantitative one, especially as classic biomarkers used clinically today to assess other organ systems are quantitative in nature.

This aspect primarily restricts the prognostic ability of a theoretical quantitative brain biomarker. There is considerable interest in the development of biochemical markers that reflect the severity of cerebral damage and correlate with short-term outcomes and long-term prognosis [7-9].

The purpose of this study was to analyze various biochemical indices, such as random blood sugar, hemoglobin, international normalized ratio (INR), lactate dehydrogenase (LDH), and cortisol, in conjunction with CT findings, to ascertain if they could identify those at risk for adverse outcomes.

## Materials and methods

This prospective observational study was conducted between 2017 and 2020 in the Department of Neurosurgery, Sri Ramachandra Institute of Higher Education and Research (SRIHER), Porur, Chennai after obtaining permission from the Institutional Ethics Committee, SRIHER (approval number: CSP-MED/19/APR/52/44). The study included 61 patients admitted to the Department of Neurosurgery with moderate and severe traumatic brain injury (TBI) who met the inclusion and exclusion criteria. The course of treatment in the hospital and the outcome were studied.

The inclusion criteria were patients with TBI having a Glasgow Coma Scale (GCS) score of 12 or less and the exclusion criteria were patients who were not willing to be treated as inpatients as well as those with significant injuries to other systems that required intervention.

Upon admission, patients were evaluated to assess GCS score, pupil size, and other neurological deficits. A simultaneous examination was carried out to rule out major systemic injuries. A CT scan was performed and classified based on the Rotterdam scoring system, as shown in Table 1 [5]. Blood was drawn to measure hemoglobin, blood sugar, INR, lactate dehydrogenase (LDH), and serum cortisol. LDH was measured in units per liter (U/L) with a normal range of 100-190 U/L. The reference range for serum cortisol was 4.3-23 mcg/dL. Patients were managed based on their injuries and those requiring surgery were operated on. They were treated in the ICU. Serum cortisol and serum LDH values were repeated on Day 5. Management was directed at controlling raised intracranial pressure (ICP).

**Table 1:** Rotterdam CT Scoring System To the calculated score, an additional 1 point was added bringing the score's range between 1 and 6.

Basal Cisterns:	
Normal	0
Compressed	1
Absent	2
Midline Shift	
<5mm	0
>5mm	1
Epidural Mass Lesion	
Present	0
Absent	1
IVH/SAH	
Absent	0
Present	1

Outcomes

Outcomes were assessed based on Glasgow Outcome Scores (GOS), elaborated in Table 2, at the time of discharge and at six months after discharge. GOS groups patients into five categories:

**Table 2:** Glasgow Outcome Scores.

GOS	OUTCOME
1	Dead
2	Vegetative state
3	Severe disability
4	Moderate disability
5	Good recovery

Statistical analysis

All statistical analyses were performed using SPSS v. 17 Windows. The data were not normally distributed and therefore non-parametric tests were performed. Descriptive statistics were presented as numbers and percentages. The data were expressed as mean and standard deviation (SD). A chi-squared test was used for the comparison between the two attributes. Multiple logistic regression was used to predict the independent variables. A two-sided p-value < 0.05 was considered statistically significant.

## Results

Demographics and presentation

The demographic data of our population are presented in Table 3. 

**Table 3:** Demographic data of the study population GCS: Glasgow Coma Scale

Patient characteristics	N=61	Percentage
Age (in years)		
<20	6	9.80%
20-40	34	55.73%
40-60	14	22.95%
>60	7	11.52%
GCS		
9-12	15	25%
6-8	18	30%
<5	28	45%
Management		
Surgical group	22	36.10%
Surgical group mortality	7	31.80%
Conservative group	39	63.90%
Conservative group mortality	17	43.60%

Of the 61 patients, 53 (86.89%) were men and the other eight (13.11%) were women. Twenty-one of the men (39.6%) and three of the women suffered a poor outcome. There was no statistical correlation between outcome and gender.

The youngest patient in our study was four years old and the oldest was 75 years old. The mean age was 38.49 ± 17.5 years. There was a trend towards poor outcomes with increasing age. Of the seven patients older than 60 years, six had a poor outcome; in contrast, only 10 of the 40 who were younger than 40 years had a poor outcome. There was a statistically significant correlation between poor outcomes and age, with a p-value of 0.004.

With regards to GCS, and outcome, there was a strong correlation between poor outcome and low GCS. Nearly two-thirds of the patients (n=18) with a GCS of 5 and less expired while six (18.18%) with a GCS of less than 8 died. The p-value for the correlation was statistically significant at 0.01. 

Twenty-two patients (36%) required surgical management, while the remaining 39 (64%) were conservatively managed. Although the mortality was high in the conservative group (17 vs. 7, 43.6% vs. 31.8%), there was no statistical correlation between the line of management and outcome. Acute subdural hematoma was the most common cause of surgical intervention (11 patients). Six patients had intraparenchymal bleeds requiring surgery with four patients with an extradural hematoma and a case of depressed fracture completing the surgical group.

Biochemical markers

Random blood sugar (RBS) values were analyzed in all patients. The mean value was 196.18 mg/dL, with a range of 85 to 564 mg/dL. The mean RBS in those who did not survive was 259.58 mg/dL, whereas in those who survived the value was 158.48 mg/dL with the outcomes tabulated in Table 4. Analysis indicated that patients with high RBS value on admission had a higher risk of mortality (p=0.000). Although serial monitoring was done and optimization was carried out with the help of the endocrinology team, only the RBS value upon presentation was analyzed in our study. We noticed that the average insulin requirement was higher in those who had a poor outcome compared to those who had a good outcome (62.6 U/day vs 22.2 U/day), implying complex neuroendocrine mechanisms at play.

**Table 4:** Outcome with respect to sugar values. RBS: random blood sugar

RBS (mg/dL)	Survivors (n=37)	Deceased (n=24)
<180	18	1
>180	19	23

The range of international normalized ratio (INR) was 0.93 to 3.43, with a mean of 1.18±0.35. The mean INR value in the recovered group was 1.08±0.11 and in the mortality group was 1.21±0.29. INR had a negative correlation with outcome (p=0.019), i.e. patients with INR >1.3 had an increased risk of mortality. None of our patients were on anticoagulants.

Hemoglobin was assessed in all patients. The minimum value was 6.2 g/dL and the maximum was 18.5 g/dL, with a mean of 12.86 ± 2.29 g/dL. The mean hemoglobin of patients in the mortality group was 13.03 ± 2.18 g/dL and in those who recovered, it was 12.86 ± 2.8 g/dL. There was no statistically significant difference between hemoglobin levels on admission and outcome (p=0.687).

Serum cortisol levels in various groups are presented in Table 5. We noted that the mean serum cortisol levels on both Days 1 and 5 were higher in patients who died and were able to establish a statistically significant correlation between both the values and outcome. Although there was a trend towards increasing serum cortisol levels and worse GCS, we were not able to establish a statistically significant correlation (p=0.07).

**Table 5:** Serum cortisol values on Days 1 and 5 in various groups. GCS: Glasgow Coma Scale

Serum Cortisol	Day 1 (mcg/dL)	Day 5 (mcg/dL)
Mean Value	24.98 ± 4.62	22.77 ± 5.48
Mean Value in Recovery group	24.04 ± 1.39	21.29 ± 1.93
Mean Value in Deceased group	29.26 ± 4.20	28.68 ± 4.03
Mean Value in GCS of 5 and less	31.49 ± 4.87	25.96 ± 3.90
Mean Value in GCS 6-8	25.88 ± 5.21	23.14 ± 2.96
Mean Value in GCS 9-12	16.71 ± 3.59	19.36 ± 4.62

With respect to the change in levels of cortisol between Days 1 and 5, we found that patients who had an increase in serum cortisol between Days 1 and 5 had a poor prognosis. This was corroborated statistically with a p-value of 0.015.

Serum lactate dehydrogenase (LDH) levels in various groups are presented in Table 6. A statistically significant negative correlation between Day 1 and Day 5 serum LDH levels and outcome was evident from our study (p = 0.000 for both). Day 1 serum LDH levels had a negative correlation with GCS on admission (p = 0.008), while Day 5 serum LDH levels were higher in patients with lower GCS, there was no correlation with GCS.

**Table 6:** Serum LDH values on Days 1 and 5 in various groups LDH: lactate dehydrogenase; GCS: Glasgow Coma Scale

Serum LDH	Day 1 (U/L)	Day 5 (U/L)
Mean Value	367.38 ± 58.49	463.31 ± 23.84
Mean Value in the Recovery group	364.20 ± 27.04	416 ± 31.80
Mean Value in the Deceased group	419.28 ± 48.23	726 ± 56.42
Mean Value in GCS of 5 and less	549.91 ± 56.73	561.67 ± 62.91
Mean Value in GCS 6-8	352.33 ± 44.72	365.54 ±39.41
Mean Value in GCS 9-12	382.06 ± 48.35	464.75 ± 34.82

With respect to the change in levels of LDH between Days 1 and 5, we found statistical corroboration between an increase in LDH levels and outcome (p=0.009). In patients who had an increase in both serum cortisol and LDH between Days 1 and 5, there was a trend toward poor outcomes (p=0.00). 

Of all the patients who succumbed to the injury (n=24), 17 (70.83%) had an increase in both cortisol and LDH values between Days 1 and 5. Of the 30 who had an increase in both LDH and cortisol between Days 1 and 5, 56.67% (n=17) did not survive. This trend had a statistically significant p-value of 0.000.

Radiological factors

Acute subdural hematoma (ASDH) was the commonest type of bleed noted in 26 patients (42.62%), followed by 17 traumatic ICH (27.86%). Diffuse axonal injury (DAI) was the third most common CT diagnosis seen in nine patients (14.75%). Seven cases of epidural hematoma (EDH) (11.47%) and one case each of brainstem contusions and depressed fracture completed our series. ASDH had the highest mortality with 15 patients dying (57.69%) followed by four cases of intracerebral hemorrhage (ICH) (23.52%). Serum cortisol levels and serum LDH levels on Day 1 and Day 5 had no association with CT findings or severity.

The distribution of patients according to the Rotterdam CT scoring system is presented in Table 7. A significant negative correlation was noted between the Rotterdam score and outcome (p-value=0.00). There was also a correlation between the score and the GCS at presentation. Among the components of the Rotterdam score, the presence of IVH in CT scan had a significant association with unfavorable outcomes (p=0.01) while midline shift was significantly associated with a low GCS (p=0.04).

**Table 7:** Rotterdam CT score and patient distribution

Rotterdam CT Score	Number (n=61)	Percentage (%)	Mortality (n=24)	Percentage (%)
1	5	8.2%	0	0
2	14	22.95%	2	14.28%
3	10	16.4%	2	20%
4	9	14.75%	2	22.2%
5	21	34.4%	14	66.67%
6	8	13.11%	4	50%

We were not able to establish any significant correlation between the imaging findings and the biochemical markers we had studied.

Outcomes

Patients were followed up in person or through telephonic conversation and GOS was assessed at discharge and at six months. With respect to mortality, 24 patients in our study died (39.34%), while the remaining patients had various degrees of recovery. During the follow-up period, we noted five patients improve from GOS 4 to GOS 5 while one patient improved from GOS 3 to GOS 4 implying the importance of rehabilitation (Table 8). 

**Table 8:** Outcome Assessment GOS: Glasgow Outcome Score

GOS	OUTCOME	At Discharge	At 6 months
1	Dead	24	24
2	Vegetative State	0	0
3	Severe Disability	5	4
4	Moderate Disability	9	5
5	Good Recovery	23	28

In the group with GCS ≤5 (n=28), the mean GOS was 2.21. In the group of patients with a GCS of 6-8 (n=18), the mean GOS was 3.67. In the group of patients with GCS of 9-12 (n=15), the mean GOS was 4.60. GCS on admission had a significant association with GOS at six months (p=.000)

Among the biochemical markers that were studied, Day 1 and Day 5 serum LDH values and cortisol levels, sugar values, and INR on admission all had a statistically significant association with GOS at six months (p< 0.05). An increase in serum cortisol individually and in concurrence with S. LDH levels between Days 1 and 5 also had a statistically significant correlation with outcomes. Rotterdam score had a statistically significant association with GOS at six months (p=0.001). A summary of factors with a statistically significant correlation with outcome is mentioned in Table 9. 

**Table 9:** Correlation of various parameters with outcomes LDH: lactate dehydrogenase; IVH: intraventricular hemorrhage; GCS: Glasgow Coma Scale

Parameter	p-value with respect to outcome
LDH levels	0.0
Cortisol levels	0.021
Increase in cortisol/LDH levels between Days 1 and 5	0.015/0.009
Increase in LDH and cortisol between Days 1 and 5	0.000
Random blood sugar	0.0000
INR	0.019
Rotterdam CT Score (IVH)	0.00 (0.01)
GCS	0.01

## Discussion

Age is a strong prognostic factor in traumatic brain injury (TBI) [10]. Various studies have noted that increasing age is associated with poorer outcomes. There was a significant association of unfavorable outcomes with age > 40 years in patients presenting with GCS less than 12 in a study published by Dhandapani et al. in 2012 [10]. The results of our study are similar; the mean age of those who died (56.8 years) was higher than the mean age of those who recovered (38.5 years). We also found that of the seven patients older than 60 years, six had a poor outcome while in contrast, only 10 of the 40 who were younger than 40 years had a poor outcome. The presence of comorbidities and atherosclerotic cerebrovascular disease increases the risk of secondary insult while decreased free radical scavenging may increase oxidative damage [11].

Kraus et al. [12] concluded that the mortality rate of women compared to men suffering from TBI was 1.28 times higher on average. Women were 1.57 times more likely to suffer from post-traumatic symptoms than men. Farace et al. [13] showed that in 17 out of 20 studies, which analyzed the effect of sex on TBI outcomes, women suffered worse overall from TBI events. In our study, there was no difference in outcome based on gender. This is probably because women constituted a minority of our study population and prevalent social issues often prevent women from accessing higher medical care.

In 1974, Teasdale and Jennett [14] introduced the Glasgow Coma Scale (GCS) to assess the severity of TBI. Many studies have validated the usefulness of GCS in identifying the severity of injury and as an indicator of outcome. Patients with GCS less than or equal to 5 had the highest mortality (66.66%). Mortality in the severe TBI group (GCS <8) was 51.16%. A strong correlation between GCS on admission and outcome was seen in our study. Midline shift had a significant correlation with admission GCS (p=.024); patients with a midline shift of > 5 mm had lower GCS on arrival. GCS also had a strong association with GOS at six months (p=.000). GCS also had a statistically significant negative correlation with the Rotterdam score, making GCS one of the most important tools in the assessment of TBI. 

Studies have demonstrated that admission hyperglycemia was associated with adverse outcomes [15,16]. Falkowska et al., [17] in a study on 267 patients with moderate or severe TBI, demonstrated that the levels of hyperglycemia were reliable predictors of severity and neurological damage. In our study, the mean RBS in those who recovered was 157.68 ± 58.41mg/dL, and in those who died was 259.58 ± 131.90.mg/dL. High RBS levels were associated with increased mortality and were found to be statistically significant (p=0.000).

A systematic review of 34 studies over a 40-year period by Corbett et al. from Western Australia [18] found an overall incidence of coagulopathy in as many as 60% of patients with severe TBI. More importantly, the presence of coagulopathy after TBI was reported to be associated with a dramatic increase in mortality compared to those without (46% vs. 7%). Greuters et al. [19] found coagulopathy, both on arrival and during the first 24 hours, an independent prognostic factor for unfavorable outcomes. In our study, we found admission INR has a negative correlation with outcomes (p=0.019), implying that deranged coagulation can complicate management - especially surgery - and can lead to poor outcomes. This offers a role for agents like tranexamic acid in the management of TBI which can cause disturbances in coagulation.

Elevated serum cortisol levels have been previously reported in patients with mild and moderate TBI [20,21]. Additionally, a positive correlation between the severity of the injury and cortisol concentration has been shown in patients with mild or moderate TBI, but not in those with severe injury [22]. Wagner et al. [23] evaluated serum cortisol levels serially from Day 0 to Day 6 of TBI. There were three distinct group profiles identified for serum cortisol: a high, decliner, and low group. Although cortisol levels for each group were significantly different from each other for all seven days, it had no influence on the outcome. Studies have suggested that several factors cause a rise in cortisol levels through neuroendocrine stimulation such as direct stress and indirect inflammatory response all of which act on the hypothalamic-pituitary-adrenal axis [20]. 

Rao et al. [24] demonstrated a statistically significant positive correlation between serum cortisol and GCS levels. The mean cortisol levels estimated were significantly increased in mild head injury and a greater increase was seen in cases of moderate and severe head injuries. Salehpoor et al. [15] found that serum cortisol had no strong relation with the outcome of patients with brain injury, but our study demonstrates a significant association between elevated cortisol on Day 1 as well as Day 5 and outcome at discharge. An increase in cortisol between Days 1 and 5 was associated with worse outcomes. A mean trend was a decrease between Days 1 and 5, only a marginal fall was noted in those with GCS of less than 9 compared to those with higher GCS who had a mean increase in cortisol levels. This may represent the complex balance between pro and anti-inflammatory pathways in those with a head injury. 

Lactate dehydrogenase is a cytosolic enzyme present in the brain that, in response to injury, is released into the bloodstream in quantities appropriate for the degree of injury. In 1983, Bakay and Ward [25] reported the results of serum LDH measurements in 49 patients with severe head injury (GCS score of 3-7) and 30 patients with moderate head injury. They found no correlation between LDH levels and outcome. However, a recent study by Bhardwaj et al. [26] showed that LDH value can differentiate the patients with respect to outcome. 

According to Jain et al. [27], serum LDH activity estimation with clinico-radiological evaluation can help in predicting the outcome of head injury and also be helpful in decision-making on further management. Cohan et al. [28] analyzed various factors and found that only GCS, glucose, and LDH were statistically significant risk factors for mortality. In our study, Serum LDH (on Days 1 and 5) was able to predict outcome (p=0.000). The difference in value between Days 1 and 5 was highest in groups with poor outcomes compared to those who recovered. This probably denotes the extent of damage that had occurred on Day 1 and ongoing injury making LDH a significant predictor of poor outcome.

Deepika et al. [29] compared the Marshall and Rotterdam CT scan scoring systems and found both effective in predicting early mortality in moderate and severe traumatic brain injury. In our study, the Rotterdam system was able to predict mortality with higher grades pointing towards increased mortality. Various studies showed that separate components like the presence of traumatic SAH/IVH and midline shift had independent prognostic value. When we analyzed those factors independently, IVH had a significant correlation with mortality (p=0.001) while midline shift had a significant correlation with GCS. 

We also noted improvement in outcomes in patients who were GOS 3 and GOS 4 at the time of discharge. This underscores the importance of early and aggressive rehabilitation. 

Limitations of the study

Since patients with systemic injuries were excluded from the study and only patients with moderate and severe head injuries were studied, our sample size was only sixty-one.

## Conclusions

Biochemical markers such as INR, RBS, serum cortisol, and LDH values at the time of admission can serve as useful adjuncts in patients with traumatic brain injury. Simultaneous increases in both LDH and cortisol levels between Days 1 and 5 are good indicators of mortality.

The Glasgow Coma Scale continues to be an indispensable tool for assessing and evaluating patients with TBI. It has a good correlation with the Rotterdam scoring system, which independently serves as a useful adjunct to predict outcomes in TBI. The presence of IVH is an independent poor prognostic factor in predicting outcomes.

Based on these parameters, one can identify those at risk of poor outcomes as well as those who are likely to recover to initiate appropriate management plans, including early and aggressive rehabilitation services, which play a significant role in the improvement of patients with TBI. 
